# Editorial: Advancements in cognitive-linguistic rehabilitation of post-brain injury: mechanisms and strategies

**DOI:** 10.3389/fneur.2026.1775280

**Published:** 2026-02-06

**Authors:** Liu Rui, Zhao Yuting

**Affiliations:** Department of Rehabilitation, The Second Affiliated Hospital of Air Force Medical University, Xi'an, Shannxi, China

**Keywords:** aphasia therapy, behavioral techniques, brain injury, cognitive rehabilitation, neurorehabilitation, speech impairment

## Introduction

Cognitive and linguistic impairments are among the most common and impactful consequences of brain injury, which directly or indirectly impact the functional recovery of other abilities in patients. Severe cognitive deficits prevent patients' participation in targeted rehabilitation. Meanwhile, profound linguistic impairments often occur with hand motor dysfunction and swallowing disorders, significantly impairing comprehensive rehabilitation. Yet, critical questions remain regarding the mechanisms underlying rehabilitation, the effectiveness of various intervention strategies, and how to translate the achieved results into clinical outcomes.

## Overview

This Research Topic covers nine high-quality studies, including basic science, clinical innovation, and translational research. It illuminates evaluations, therapeutic interventions, and investigations using electrophysiology, neuroimaging, and behavioral techniques. Collectively, these studies expand the evidence base in this field, providing emerging insights and practical directions to guide the interventions for post-brain-injury-related cognitive and linguistic impairments.

A key theme across several contributions is the relationship between brain region, functional connectivity, and cognitive impairments. One study investigated alterations in functional connectivity (FC) among thalamic subregions in patients with basal ganglia stroke (Cheng et al.). By using resting-state functional magnetic resonance imaging, results showed that basal ganglia stroke disrupts thalamocortical circuitry at subregional levels, with left PFC-thalamus-right supramarginal connectivity predicting MMSE performance. These network-level insights provide potential biomarkers for monitoring recovery and personalizing interventions to improve cognitive outcomes in stroke patients. A second study assessed the effects of high-frequency repetitive transcranial magnetic stimulation (rTMS) in patients with post-stroke cognitive impairment (PSCI) (Song et al.). In this work, transcranial magnetic stimulation-electroencephalography (TMS-EEG) was applied to record functional connectivity. Results demonstrated that rTMS effectively modulated interregional connectivity between the left dorsolateral prefrontal cortex (DLPFC) and associated networks and pointed to the crucial role of the DLPFC in cognitive functions.

Early detection are essential to advance intervention and prognosis of cognitive and linguistic disorders. This Research Topic further included studies focusing on biomarker discovery. A meta analysis by Wang et al. explored the relationship between malnutrition and cognitive deficits. They pointed out that the period from stroke onset to PSCI development is a critical window for preventing cognitive impairment. The study found that decreased serum albumin levels and abnormal albumin-related nutritional indices are significantly associated with an increased risk of PSCI. This work provides clinically accessible indicators for early PSCI risk stratification. In addition, Gu et al. systematically profiled lncRNA expression in post-stroke aphasia (PSA), identifying 797 differentially expressed lncRNAs in peripheral blood mononuclear cells. In this study, lncRNA RP11-227G15.3 was significantly upregulated and negatively correlated with oral spelling ability. This pioneering study suggests that lncRNAs may serve as a potential biomarker for monitoring cognitive improvement.

For PSA, cortical area has long been regarded as the main damage source, while growing evidence shows that subcortical damage can also cause significant language deficits. An original research study by Yuan et al. systematically characterized the language profiles and prognosis of patients with subcortical aphasia. Four standard aphasia batteries were used and 1-year follow-up assessment was conducted in this study. It identified two core impairment dimensions in these patients: lexical-semantic processing and phonological-auditory processing. These dimensions can effectively predict aphasia severity and prognosis. The study provides more detailed insights into language processing deficits.

Alzheimer's disease (AD) represents a degenerative condition affecting the central nervous system, mainly presenting with cognitive impairment. This Research Topic includes a systematic review by Yan et al. that clarifies the complex relationship between gait patterns and cognitive status in AD patients. The review found that gait parameters (including speed, variability, and rhythm) were closely associated with cognitive function and may serve as surrogate biomarkers for cognitive decline.

In addition to biomarker discovery, this Research Topic contains several meta-analyses evaluating the effects of cognitive and linguistic interventions. Traditional cognitive rehabilitation typically involves therapist-led interventions, which are either implemented individually or in group settings, often with the support of multidisciplinary teams. Recently, technology-based interventions have become an increasingly viable alternative to traditional therapy. Chi et al. presented a meta-analysis to evaluate the effects of digital cognitive intervention on cognitive function. Results showed that VR-based interventions exhibited superior effects to traditional cognitive therapy, which significantly improved cognition, executive function, attention, and social cognition in traumatic brain injury (TBI) patients (Chi et al.).

Further, a meta-analysis by Liang et al. assessed the efficacy of non-invasive brain stimulation (NIBS) techniques in patients with attention-deficit/hyperactivity disorder (ADHD). Their work revealed that that dual tDCS (anodal left DLPFC + cathodal right supraorbital) optimized cognitive flexibility in ADHD patients. Meanwhile, bilateral DLPFC stimulation enhances working memory.

Besides, a protocol by Jia et al. outlines a planned systematic review and meta-analysis. It aims to synthesize evidence on the effectiveness of acupuncture for PSA patients. This study will evaluate its impact on language function, functional communication, and quality of life, with the aim to establish conclusive evidence for the standardized application of acupuncture in neurorehabilitation.

Collectively, this Research Topic's contributions cover multiple neurological disorders, including stroke, AD, ADHD, and TBI, focusing on biomarker discovery, therapeutic interventions, and assessment tool development. These studies provide a comprehensive overview in cognitive-linguistic rehabilitation mechanisms and clinical applications.

## Future research directions

The findings highlight remaining challenges. Firstly, to develop practical early screening and prognostic tools, future research should prioritize clinical candidate biomarkers. It is particularly important to verify their stability and specificity in large-scale, multi-center longitudinal cohorts. Second, with the development of artificial intelligence technology, future research should integrate multimodal technologies to enhance the accuracy and detection efficiency of the assessment, intervention and monitoring process for cognitive and language disorders, promoting intelligent rehabilitation.

